# A novel systemically administered toll-like receptor 7 agonist potentiates the effect of ionizing radiation in murine solid tumor models

**DOI:** 10.1002/ijc.28711

**Published:** 2014-01-17

**Authors:** Amy L Adlard, Simon J Dovedi, Brian A Telfer, Erina Koga-Yamakawa, Charlotte Pollard, Jamie Honeychurch, Timothy M Illidge, Masashi Murata, David T Robinson, Philip J Jewsbury, Robert W Wilkinson, Ian J Stratford

**Affiliations:** 1 Experimental Oncology Group, School of Pharmacy and Pharmaceutical Sciences, Manchester Cancer Research Centre, University of Manchester, Manchester Academic Health Sciences CentreUnited Kingdom; 2 Targeted Therapy Group, Institute of Cancer Sciences, Manchester Cancer Research Centre, University of Manchester, Manchester Academic Health Sciences CentreUnited Kingdom; 3 Dainippon Sumitomo PharmaKonohana-ku, Osaka, Japan; 4 AstraZeneca Pharmaceuticals Ltd.Alderley Park, Cheshire, United Kingdom

**Keywords:** TLR7, immunotherapy, radiotherapy, combination therapy, murine tumor model

## Abstract

Although topical TLR7 therapies such as imiquimod have proved successful in the treatment of dermatological malignancy, systemic delivery may be required for optimal immunotherapy of nondermatological tumors. We report that intravenous delivery of the novel small molecule TLR7 agonist, DSR-6434, leads to the induction of type 1 interferon and activation of T and B lymphocytes, NK and NKT cells. Our data demonstrate that systemic administration of DSR-6434 enhances the efficacy of ionizing radiation (IR) and leads to improved survival in mice bearing either CT26 or KHT tumors. Of the CT26 tumor-bearing mice that received combined therapy, 55% experienced complete tumor resolution. Our data reveal that these long-term surviving mice have a significantly greater frequency of tumor antigen specific CD8^+^ T cells when compared to age-matched tumor-naïve cells. To evaluate therapeutic effects on spontaneous metastases, we showed that combination of DSR-6434 with local IR of the primary tumor significantly reduced metastatic burden in the lung, when compared to time-matched cohorts treated with IR alone. The data demonstrate that systemic administration of the novel TLR7 agonist DSR-6434 in combination with IR primes an antitumor CD8^+^ T-cell response leading to improved survival in syngeneic models of colorectal carcinoma and fibrosarcoma. Importantly, efficacy extends to sites outside of the field of irradiation, reducing metastatic load. Clinical evaluation of systemic TLR7 therapy in combination with IR for the treatment of solid malignancy is warranted.

**What's new?:**

Recent evidence suggests that damage from ionizing radiation (IR) can render tumor cells immunogenic. Unfortunately, established tumors often suppress this anti-tumor immune response. Combination therapy with IR and immune-modulators such as Toll-like-receptor (TLR) family agonists may overcome this problem. In this proof-of-concept study, the authors examined one such small-molecule drug, called DSR-6434. They found that systemic administration of DSR-6434 can enhance the effectiveness of radiotherapy in mice, and that this occurs via the generation of tumor-specific immune responses. Easily delivered drugs that activate TLR-family molecules may thus offer a promising therapeutic approach.

## Introduction

Radiation therapy is used in the treatment of around 50% of cancer patients and remains the most important nonsurgical treatment in the management of solid malignancies.^1^–^4^ Treatment with ionizing radiation (IR) induces lethal DNA damage leading to cellular death through mitotic catastrophe, necrosis and apoptosis. Recent evidence also suggests that treatment with IR can render the tumor cells immunogenic and potentially generate antitumor immune responses.^5^,^6^

Exposure of cancer cells to IR leads to cellular stress and the expression of several damage-associated molecular patterns (DAMPs). These include High Mobility Group Box 1 (HMGB1) and the extracellular release of ATP, which can activate professional antigen presenting cells (APCs), such as the dendritic cell (DC), and engender tumor antigen-specific T-cell responses.^6^,^7^ In addition, the induction of DNA damage subsequent to treatment with IR can lead to the production of novel (and potentially immunogenic) proteins and has been shown to upregulate tumor cell expression of class I MHC.^5^ Taken together, these data demonstrate the immunogenic potential of radiation therapy for cancer. Data from preclinical studies, however, demonstrate that established tumors foster immunosuppressive microenvironments that favor angiogenesis and the production of cytokines such as transforming growth factor-β (TGF-β) and interleukin-10 (IL-10),^8^ which attenuate T_H_−1 cytotoxic activity.^9^ Consequently, in both preclinical and clinical studies the use of IR alone is rarely capable of generating durable tumor antigen-specific immune responses.^10^,^11^ However, several preclinical studies have demonstrated the generation of systemic immune responses following combination therapy with IR and immuno-modulators such as monoclonal antibodies to CTLA4 and CD40, and small molecule agonists of the Toll-like receptor (TLR) family members TLR7 and TLR9.^11^–^14^

Members of the TLR family are responsible for recognizing a diverse array of evolutionarily conserved pathogen-associated molecular patterns and are constitutively expressed by both professional APCs and effector immune cell populations.^15^–^18^ Activation of TLR7, which is localized intracellularly in endosomal membranes and recognizes viral guanosine and/or uridine-rich single-stranded RNA,^19^–^21^ leads to a MyD88-dependent signaling cascade ultimately resulting in interferon regulatory factor-7 (IRF-7), AP-1 or NFκB-mediated transcription of T_H_1 cytokines, predominantly Type I interferon (IFN).^22^

Synthetic small molecule agonists of TLR7 have been developed such as imiquimod (Aldara 5% cream, 3M), which is FDA-approved for the treatment of genital warts and superficial basal cell carcinoma. However, delivery of topical TLR7 agonists to noncutaneous tumors is challenging and would require image-guided delivery such as ultrasound or computerized tomography. We have previously demonstrated that systemic delivery of a TLR7-selective small molecule agonist leads to the priming of systemic immune responses capable of reducing both primary and metastatic tumor burden.^23^ The TLR7 agonist 852A (Pfizer) is currently one of the most extensively studied compounds to be tested systemically in clinical trials.^24^ In the present study we assess the antitumor efficacy of a newly described systemically administered 8-oxoadenine derivative, DSR-6434, which has a higher water solubility and a 300-fold greater potency for human TLR7 than 852A.^25^ Our results demonstrate that systemic administration of DSR-6434 can enhance the effectiveness of radiotherapy in two syngeneic tumor models and that this occurs as a consequence of TLR7 activation and the generation of tumor-specific immune responses.

## Materials and Methods

### Mice, cell lines and reagents

Animal experiments using 8–12-week-old female C3H mice (bred in-house at the University of Manchester UK), BALB/c (Clea, Japan and Harlan Laboratories, UK) and TLR7 knockout (−/−) BALB/c (Oriental Yeast, Japan) mice were conducted according to the guidelines of the Animal Care and Use Committee at Dainippon Sumitomo Pharma, Japan; or in accordance with the UK Home Office Animal (Scientific Procedures) Act 1986 and the University of Manchester Ethics Committee, using guidelines outlined by Workman *et al*.^26^ (PPL number: 40/3212, P.I: Kaye Williams). CT26 murine colon carcinoma cells (ATCC) were maintained in Dulbecco's Modified Eagle's Medium (Invitrogen, UK). The KHT murine sarcoma cell line was produced from viable tumor pieces as described previously^27^ and was maintained in RPMI-1640 medium (Invitrogen, UK). Both growth media were supplemented with 10% (v/v) FBS (Biosera, UK) and 1% (v/v) l-glutamine (Invitrogen, UK). Cell lines were used at passage numbers of less than 12, and were regularly screened for the presence of Mycoplasma (PlasmoTest; Invivogen, UK). DSR-6434 was dissolved in 10% (v/v) DMSO for a stock solution of 1 mg/mL, and diluted in physiological saline before administration by intravenous (i.v.) injection at doses of 0.01 or 0.1 mg/kg.

### Analysis of cytokines by ELISA

BALB/c wild-type (WT) and TLR7^−/−^ mice were given 0.1 mg/kg DSR-6434 i.v. in saline (adjusted to pH5). Blood samples were taken 2, 4, 8, 12 and 24 hr after administration, and plasma samples were collected following centrifugation at 2486 *g* for 10 min at 4 °C. Baseline levels were also determined using plasma samples from untreated mice from each group. IFNα and IP-10 were measured by enzyme-linked immunosorbent assay (ELISA) or Milliplex assay in accordance with manufacturer's protocol (PBL Interferon Source and Millipore, respectively) as suitable biomarkers for TLR7 activation.^28^

### TLR7, TLR8 and TLR9 reporter gene assay

TLR7, TLR8 and TLR9 reporter gene assays were performed in HEK293 cells as described previously.^23^ The TLR7/TLR8 agonist, R-848, or the TLR9 agonist, CpG oligodeoxynucleotide (ODN) 2006 (Invivogen), were used as positive controls.

### Analysis of splenocyte activation

Mice bearing tumors with a volume of 180–220 mm^3^ received 0.1 mg/kg DSR-6434 or saline (i.v.), and spleens were harvested 4 hr later. Splenocytes were isolated using a 90 µm cell strainer, red blood cells were lysed in Pharm Lyse™ (BD Biosciences, UK) and incubated with αCD16/CD32 Fc blocking antibody (eBioscience) followed by CD49 pan NK-FITC (5 µg/mL), CD69-PE (2 µg/mL), CD3ε-PECy5 (2 µg/mL; all from BD Pharmingen) and CD19-FITC (5 µg/mL; eBioscience). Samples were analyzed by flow cytometry (FACScanto, BD Biosciences) and analyzed as previously described.^14^

### Clonogenicity assays

Exponentially growing CT26 and KHT cells were plated at densities of 1 × 10^2^ to 2.4 × 10^3^ cells/well. 24 hr later, cells were treated with a range of concentrations of DSR-6434, followed 2 hr later by treatment with 4 Gy X-ray radiation. Media were replaced 24 hr later, and cells were left to proliferate for ∼7 days or when colonies of more than 50 cells were visible in the untreated wells. Colonies were stained with 0.5% (w/v) methylene blue solution and counted manually. Surviving fractions were normalized to the nonirradiated controls, or when radiation was not used, the untreated control.

### MTT proliferation assay

CT26 and KHT cells were seeded at a density of 1000 and 1500 cells/well, respectively. The following day, cells were treated with varying concentrations of IFNα (PeproTech, UK). Media was replaced after 24 or 48 hr, or was not removed until the experimental endpoint at 72 hr. After 72 hr, 50 µL of 5 mg/mL 3-(4,5-dimethylthiazol-2-yl)−2,5-diphenyltetrazolium bromide (MTT) was added to wells for 4 hr, after which the media were replaced with DMSO for analysis of optical density using a µQuant Microplate Spectrophotometer (BioTek, UK) at a wavelength of 540 nm.

### Tumor therapy

Syngeneic tumors were established following subcutaneous implantation of 1 × 10^5^ CT26 cells or 5 × 10^5^ KHT cells 1 cm from the base of the tail. Tumor volume was measured using calipers as *length × width × depth*. When palpable tumors developed, mice were randomly assigned into treatment groups and treatment commenced at tumor volumes of 180–220 mm^3^. Treatment groups were saline, DSR-6434, IR, and a combination of DSR-6434 and IR. DSR-6434 was administered 4 hr before radiotherapy commenced and once weekly thereafter for a total of 4 doses, a schedule that maximizes the immune stimulation on repeat dosing in monotherapy studies. The experimental end-point was the time taken for tumors to reach 4× the volume at the time of treatment (RTV4). Thereafter the mice were sacrificed. KHT and CT26 tumors received a single dose of 25 or 15 Gy, or 5 daily fractions of 2 Gy, respectively. For irradiation, nonanesthetized mice were restrained in custom-made lead shields allowing the local irradiation of tumors. Radiation was administered using 250 kV X-rays (MXR-320/36 X-ray tube, Comet AG, Switzerland) at 12 mA and a dose rate of 2 Gy/min. Mice were considered to be long-term survivors (LTS) if they were tumor-free for greater than 90 days post-treatment.

### Analysis of intracellular IFNγ production

Splenocytes harvested from either LTS mice or control mice were cultured (3.5 × 10^6^/well) for 5 days in RPMI-1640 supplemented with 10% FCS, 100 U/mL penicillin, 100 μg/mL streptomycin, 1% l-glutamine, 50 μM 2-ME and 10 IU/mL human recombinant IL-2 in the presence of either 1 × 10^6^ CT26 cells irradiated with 50 Gy (cultured in 12-well plates), 1 µM irrelevant peptide (SIINFEKL; Anaspec, UK) or 1 µM AH-1 peptide (SPSYVYHGF; Anaspec, UK) (both cultured in 24-well plates). After 5 days in culture, cells were restimulated at a 1:1 ratio with 50 Gy-irradiated CT26 cells for 16 hr in the presence of 1 µL/mL Brefeldin A (BD Pharmingen, UK) and 100 IU/mL human recombinant IL-2 (Chiron, NL). For subsequent flow cytometry analysis, cells were incubated with rat anti-CD16/32 (eBioscience, UK) and then stained with a FITC conjugated anti-CD8α mAb. Cells were then fixed/permeabilized and stained for IFNγ expression using an APC conjugated mAb (BD Pharmingen, UK).

### Assessment of lung metastasis

Lungs from C3H mice carrying subcutaneous KHT tumors were removed and graded using a semiquantitative scale from 0 to 5 with increasing severity of metastasis, as described previously by Lunt *et al*.^27^ Groups of mice were treated with either 25 Gy alone, or with 15 Gy plus DSR-6343. Mice were sacrificed and lungs excised when the primary tumors reached RTV4 (the group given 15 Gy/DSR-6343 combination). To determine directly the effect of DSR-6343 on the development of lung metastases; those mice receiving 25 Gy alone were sacrificed at the same time after radiation treatment as the 15 Gy plus DSR-6343 group.

### Statistical analysis

Where data from two *in vivo* treatment groups are compared, Mann–Whitney tests were performed. Student's two-tailed *t-*tests were used to compare groups *in vitro* and *ex vivo*. To compare survival curves from *in vivo* experiments, Log-Rank Mantel–Cox tests were performed on Kaplan–Meier plots. Results were considered to be statistically significant if *p* < 0.05. All statistical analyses were performed using GraphPad Prism v5.00.

## Results

### DSR-6434 is a specific TLR7 agonist and intra-venous administration leads to the activation of several immune effector cell populations

**Figure 1:**
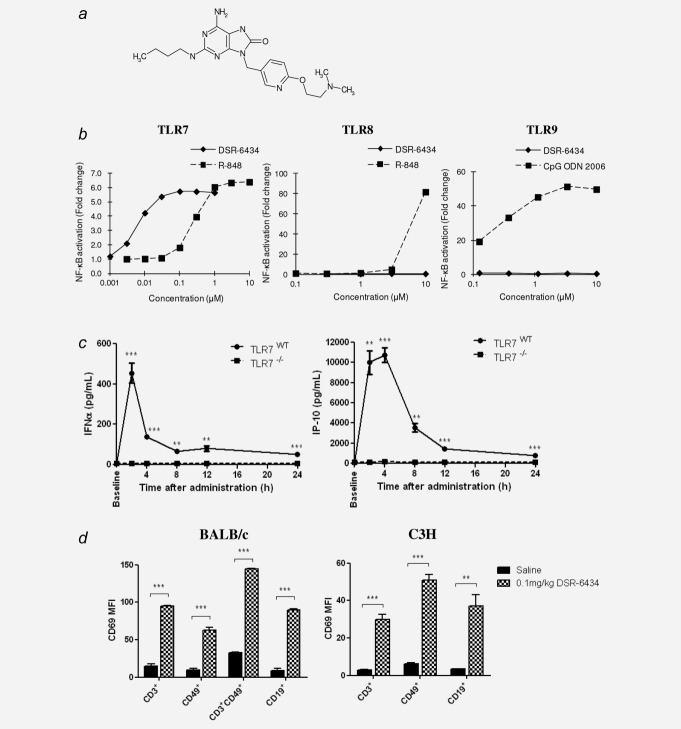
DSR-6434 is a specific TLR7 agonist and induces systemic immune activation following i.v. administration. (*a*) Chemical structure of DSR-6434. (*b*) NFκB gene reporter assays were performed in HEK293 cells using a range of concentrations (0.001–10 µM) of DSR-6434 and either R-848 or CpG ODN (2006) as positive controls. Plotted are means of *n* = 2. (*c*) BALB/c mice wild type (WT) or knockout (−/−) for TLR7 received 0.1 mg/kg (i.v.) DSR-6434. Plasma was collected at time-points between 2- and 24-hr post i.v. dose and the concentrations of IFNα and IP-10 were determined by ELISA and Milliplex assay, respectively. Data represented as mean ± SEM of three mice per group. *p < 0.05; **p < 0.01; *** *p* < 0.001 when comparing TLR7^WT^ with TLR7^−/−^ mice (two-tailed Student's *t-*test). D: Splenocytes were harvested from CT26 and KHT tumor-bearing BALB/c (left) and C3H (right) mice, respectively, 4 hr after i.v. injection with saline or 0.1 mg/kg DSR-6434. CD69 expression was analyzed by flow cytometry and plotted as mean fluorescence intensity (MFI) + SEM. Experimental groups contained three mice. **p < 0.01; ***p < 0.001, two-tailed Student's *t*-test.

To assess the specificity of DSR-6434 (structure detailed in Fig. 1*a*) toward TLR7, an NF-κB-driven reporter assay was performed in HEK293 cells engineered to express either hTLR7, TLR8 or TLR9. In this assay, successful binding of DSR-6434 to the specific receptor leads to NF-κB activation. Our data reveal that DSR-6434 was capable of stimulating reporter gene activity only in HEK293 cells expressing hTLR7 (EC_50_ = 7.9 nM) and not in HEK293 cells expressing the structurally similar hTLR8 or hTLR9 (Fig. 1*b*), demonstrating the compound's selectivity for TLR7.

*Ex vivo* stimulation of splenocytes harvested from TLR7^WT^ and TLR7^−/−^ mice with DSR-6434 (0.64–2000 nM) for 24 hr demonstrated the dose-dependent induction of several cytokines (IP-10, IL-12p70, IFNγ, KC, TNF-α) in splenocytes derived from TLR7^WT^ but not TLR7^−/−^ mice (Supporting Information Fig. 1). To confirm the capacity of DSR-6434 to activate TLR7 following i.v. administration, TLR7^WT^ or TLR7^−/−^ mice received a single i.v. dose and plasma was collected at time-points between 2 and 24 hr post-treatment (Fig. 1*c*). Peak levels of IFNα were detected 2 hr post-administration of DSR-6434 and were increased 72-fold (from <6.3 pg/mL to 455 ± 49.5 pg/mL) in the plasma of TLR7^WT^ mice (*p* < 0.001). Similarly, peak plasma levels of IP-10 were measured 4 hr post-DSR-6434 administration in TLR7^WT^ mice and were increased 148-fold (from 72.9 ± 7.9 pg/mL to 10764.6 ± 708.2 pg/mL) (*p* < 0.001). Additionally, our data demonstrate induction of KC, IFNγ and TNFα in the plasma of TLR7^WT^ mice following DSR-6434 administration (Supporting Information Fig. 2). Conversely, administration of DSR-6434 to TLR7^−/−^ mice did not significantly induce the expression of IFNα, IP-10, KC, IFNγ or TNFα at any of the time-points tested, confirming the TLR selectivity of DSR-6434.

Another biomarker of immune activation, CD69 expression, on splenic immune cell populations was assessed 4 hr after DSR-6434 administration by flow cytometry. Systemic administration of 0.1 mg/kg DSR-6434 resulted in increased CD69 expression on T cells (CD3+) by 6.5-fold, NK cells (CD49+) by 6.4-fold, NKT cells (CD3+CD49+) by 4.4-fold and B cells (CD19+) by 10.2-fold in BALB/c mice (*p* < 0.001) (Fig. 1*d*, Supporting Information Fig. 3). A similar pattern of upregulation was found in C3H mice bearing KHT tumors; however, due to the small numbers of splenic NKT cells detected in C3H mice, analysis of CD69 expression was not possible for these cells.

Taken together these data demonstrate that DSR-6434 is a TLR7 selective agonist that is capable of inducing systemic cytokine expression and activation of diverse immune cell populations following intravenous administration.

### Systemic treatment with DSR-6434 enhances the efficacy of radiation therapy in a model of colorectal carcinoma

**Figure 2:**
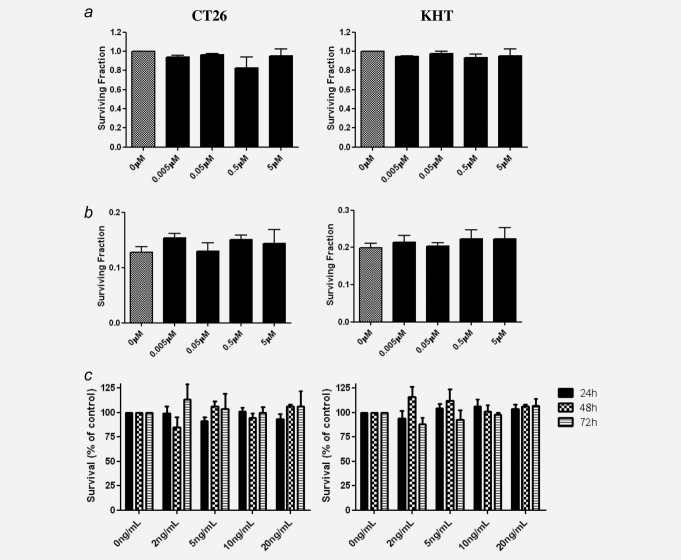
DSR-6434 does not affect tumor cell clonogenic potential or radiosensitivity, and proliferation is not affected by co-culture with IFNα *in vitro*. DSR-6434 does not affect the clonogenicity of CT26 (left panel) or KHT (right panel) cells *in vitro*, as determined by colony-forming assays. (*a*) Cells received DSR-6434 (0–5 µM) and medium was replaced 24 hr later. (*b*) Cells were treated with DSR-6434 and received 4 Gy IR 2 hr later. Medium was replaced 22 hr later. Experiments A and B ended on day 7 (CT26) or 10 (KHT), when colonies of >50 cells were present in the untreated wells. Data for 0 Gy survival fractions are normalized to 0 µM, and 4 Gy survival fraction data are normalized to the 0 Gy equivalent. (*c*) MTT proliferation assays for CT26 (left) and KHT (right) cells treated with a range of IFNα concentrations (0–20 ng/mL), with exposure times of 24, 48 and 72 hr. Data derived from three independent experiments, with means + SEM presented.

Initially, we used colony-forming assays to show that DSR-6434 was not directly cytotoxic or acting as a radiosensitizer toward CT26 or KHT tumor cells to IR-induced cell death using colony-forming assays (Figs. 2*a* and 2*b*). Cells were treated with varying concentrations of DSR-6434 from 5 nM to 5 μM alone or in combination with 4 Gy IR. No significant effect on clonogenic survival was found following treatment with DSR-6434 alone (Fig. 2*a*), nor was there a radiosensitizing effect (Fig. 2*b*). Additionally, IFNα did not affect tumor cell proliferation *in vitro*, at concentrations corresponding to those induced by TLR7 activation (Fig. 2*c*).

**Figure 3:**
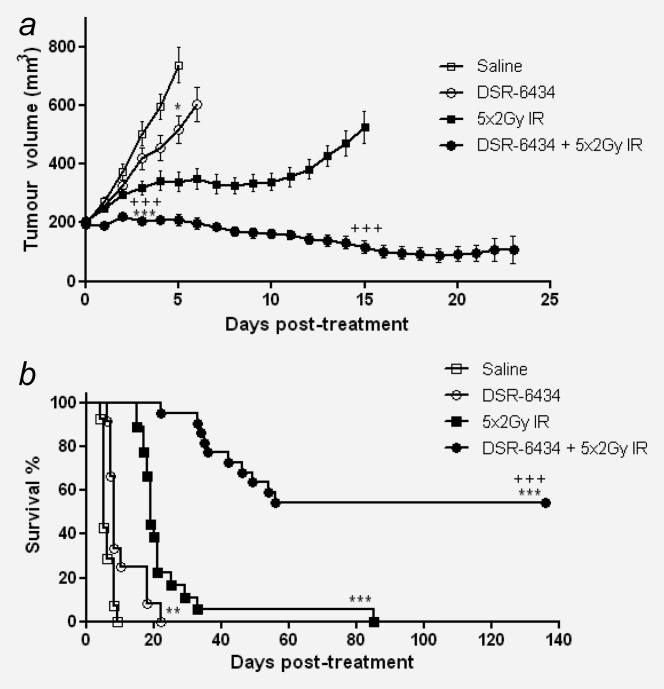
DSR-6434 potentiates the effect of radiation therapy in the CT26 model of colorectal cancer. (*a*) and (*b*) Mice received either saline (*n* = 14; open squares) or 0.1 mg/kg DSR-6434 once weekly for 4 weeks (*n* = 12; open circles) i.v., 10 Gy IR given as daily fractions of 2 Gy over 5 days (*n* = 18; filled squares) or a combination of DSR-6434 and 5 fractions of 2 Gy IR (*n* = 22; filled circles). (a) Tumor volumes are plotted from the day of therapy (day 0) until the first tumor from each group reached RTV4. Growth curves show mean ± SEM. ***/+++*p* < 0.001 relative to saline control and either monotherapy, respectively, Mann–Whitney test. (*b*) Survival curve for above cohorts. **p < 0.01 relative to saline control, +++*p* < 0.001 relative to either monotherapy, Log-rank (Mantel–Cox) test. Illustrated data combined from two identical independent experiments.

To evaluate *in vivo* efficacy, mice bearing established CT26 tumors were treated with DSR-6434 i.v. once weekly alone or in combination with 10 Gy IR delivered as 5 daily fractions of 2 Gy beginning 4 hr after the first dose of DSR-6434. Systemic administration of DSR-6434 was well tolerated when dosed weekly at 0.1 mg/kg, with no loss of condition or weight occurring as a result of mono- or combination therapy with IR (Supporting Information Fig. 4). As a monotherapy, i.v. administration of DSR-6434 at a dose of 0.1 mg/kg administered once weekly significantly reduced tumor burden when compared to vehicle-treated control mice (517.9 ± 45.8 *vs*. 736.7 ± 60.1 mm^3^; DSR-6434 monotherapy and saline-treated mice, respectively, at day 5 post-treatment) (Fig. 3*a*; *p* < 0.05). In addition, monotherapy with DSR-6434 resulted in a modest increase in survival when compared to mice receiving saline alone (RTV4 = 9.55 ± 1.3 *vs*. 6 ± 0.42 days; DSR-6434 monotherapy and saline-treated mice, respectively) (Fig. 3*b*; *p* < 0.001). Monotherapy with DSR-6434 dosed at a 10-fold lower dose of 0.01 mg/kg did not significantly impact tumor burden or affect survival (Supporting Information Fig. 5).

Treatment with 10 Gy IR delivered as 5 daily fractions of 2 Gy resulted in a substantial tumor growth delay of 17.9 ± 3.8 days relative to the saline-treated control group, and an RTV4 of 23.9 ± 3.8 (Figs. 3*a* and 3*b*; *p* < 0.001) in mice bearing CT26 tumors. Notably, 55% (12/22) of mice that received combination therapy with IR and DSR-6434 underwent complete tumor regression. Of those mice that were not able to completely reject their primary tumor, tumor delay was augmented by 16.8 ± 3.4 days relative to IR monotherapy to an RTV4 of 40.7 ± 3.4 days; (Fig. 3*b*, Supporting Information Fig. 6; *p* < 0.001). The combination of IR and DSR-6434 was found to be greater than additive since the antitumor efficacy of DSR-6434/IR combination therapy (growth delay of 34.7 days; Supporting Information Fig. 6) was considerably greater than the sum of the growth delay elicited by either monotherapy alone (21.5 days; Supporting Information Fig. 6). IHC revealed that treatment with either IR alone or IR in combination with DSR-6434 significantly increased tumor infiltration by CD8^+^ T cells when compared to size matched tumors harvested from untreated mice (*p* < 0.05; Supporting Information Fig. 7). However, no significant difference in the frequency of infiltrating CD8^+^ cells were found between cohorts that received IR alone or in combination with DSR-6434.

### Treatment with DSR-6434 and radiation therapy generates tumor antigen-specific memory T cells

**Figure 4:**
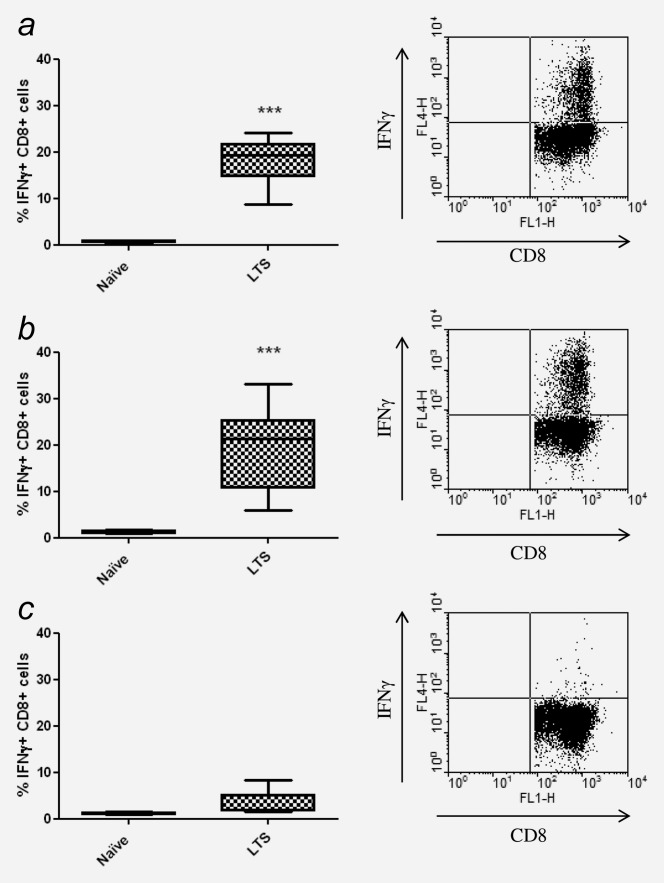
Combination therapy with IR and DSR-6434 elicits a tumor antigen-specific memory T-cell response in long-term surviving mice. (*a*)–(*c*) Splenocytes were isolated from tumor/treatment-naïve control mice (*n* = 3) and from long-term surviving (LTS) mice that had remained tumor free for greater than 90 days following DSR-6434/IR combination treatment (*n* = 5), and were co-cultured with 50-Gy-irradiated CT26 cells (*a*), AH1 peptide (*b*) or control peptide (*c*) for 6 days prior to restimulation with fresh 50-Gy-irradiated CT26 cells. The expression of IFNγ by CD8^+^ T cells was measured by flow cytometry. Representative plots of IFNγ expression by CD8^+^ T cells from LTS mice are shown to the right of each graph. ***p < 0.001, two-tailed Student's *t-*test.

The capacity of CD8^+^ T cells isolated from LTS mice originally treated with IR and DSR-6434 to produce IFNγ following co-culture with irradiated CT26 cells was determined to evaluate whether immunological memory had been established. Our data revealed that splenocytes isolated from LTS mice had a significantly greater proportion of IFNγ-producing CD8^+^ T cells following co-culture with irradiated CT26 tumor cells when compared to age-matched tumor-naïve mice (18.1 ± 2 *vs*. 0.8 ± 0.1%; LTS and naïve mice, respectively) (Fig. 4*a*; *p* < 0.001). To determine whether this response was AH1 restricted, splenocytes were also cultured with either AH1 or a control peptide for 5 days followed by restimulation with irradiated CT26 cells for 16 hr. Following co-culture with AH1 peptide the frequency of IFNγ^+^ CD8^+^ T cells from splenocytes harvested from LTS mice originally treated with IR and DSR-6434 was significantly greater than that of naïve control mice (19.9 ± 3.7 *vs*. 1.3 ± 0.1 % respectively) (*p* < 0.001; Fig. 4*b*). In contrast, no significant differences in the frequency of IFNγ^+^ CD8^+^ T cells were found between splenocytes harvested from either LTS or naïve control mice following co-culture with irrelevant peptide (3.7 ± 1.1 *vs*. 1.1 ± 0.1%, respectively) (*p >* 0.05; Fig. 4*c*). Taken together, these data demonstrate that combination therapy involving IR and DSR-6434 generates long-term tumor antigen-specific immunological memory.

### Combination therapy with local IR and DSR-6434 leads to a reduction in the incidence of lung metastasis and improved survival in the KHT fibrosarcoma model

**Figure 5:**
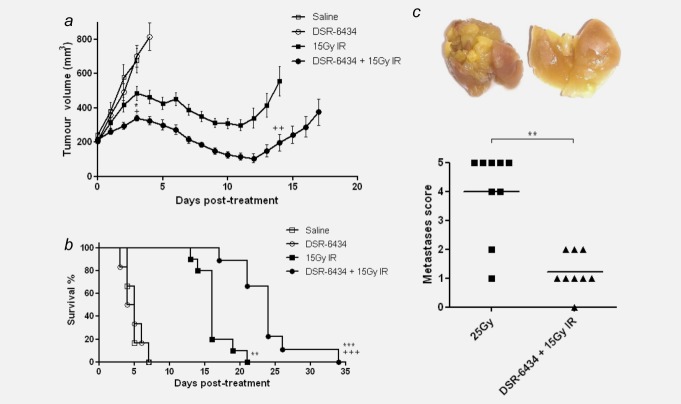
DSR-6434 potentiates the effect of radiation therapy and reduces metastatic burden in the KHT model of fibrosarcoma. (*a*) and (*b*) Mice received either saline (*n* = 6; open square), 0.1 mg/kg DSR-6434 i.v. (*n* = 6; open circle), a single dose of 15 Gy IR (*n* = 10; filled square) or a combination of 15 Gy IR and DSR-6434 (*n* = 12; filled circle). (*a*) Tumor volumes are plotted from day 1 of therapy until the first tumor from each group reached RTV4. Growth curves show mean ± SEM. + *p* < 0.05 and ++ *p* < 0.01 relative to either monotherapy, Mann–Whitney test. (*b*) Survival curve for above cohorts. **p < 0.01 relative to saline control and +++*p* < 0.001 relative to either monotherapy, Log-rank (Mantel–Cox) test. Illustrated data combined from two identical independent experiments. (*c*) Lungs were removed when primary tumors reached RTV4 (DSR-6434/15 Gy IR group) and were time-matched against mice treated with 25 Gy IR. Lung metastases were scored using a semiquantitative scoring system. **p < 0.01, Mann–Whitney test. Representative images of lungs from 25 Gy IR (left) and DSR–6434/15 Gy IR (right) treatment groups are shown above the corresponding data point.

The efficacy of DSR-6434 was assessed in the poorly immunogenic and metastatic sarcoma model, KHT.^29^ As a monotherapy, DSR-6434 given at 0.1 mg/kg had no effect on tumor burden or survival relative to the saline control, with a mean RTV4 of 4.7 days in both groups (Figs. 5*a* and 5*b*, Supporting Information Fig. 6). A single dose of 15 Gy IR delayed primary tumor growth by 11.7 ± 0.7 days relative to the saline control and significantly increased survival (*p* < 0.001; Figs. 5*a* and 5*b*, Supporting Information Fig. 6). DSR-6434 enhanced the efficacy of IR with a significant reduction in tumor volume apparent as early as 3 days from the start of treatment. At this time-point, tumors that received 15 Gy IR had a mean volume of 485.7 ± 41.8 *vs*. 340.8 ± 17.3 mm^3^ for those receiving IR/DSR-6434 combination (Fig. 5*a*; *p* < 0.05). At day 14 post-irradiation; when the first 15 Gy-treated tumor had reached RTV4, the average tumor volume in the 15 Gy IR monotherapy treatment group was 555.1 ± 86.4 *vs*. 196.2 ± 48.1 mm^3^ in the 15 Gy IR/DSR-6434 combination group (Fig. 5*a*; *p* < 0.01). Radiation-induced KHT tumor growth delay was potentiated with DSR-6434 by 7.5 days relative to the 15 Gy IR monotherapy group, with an RTV4 value of 23.9 ± 1.5 days (*p* < 0.001, Fig. 5*b*, Supporting Information Fig. 6). Combination therapy with IR and DSR-6434 led to a significant increase in survival when compared to either monotherapy alone (Fig. 5*b*; *p* < 0.001).

The KHT model is a well-characterized tumor to evaluate therapeutic effects on spontaneous lung metastases.^27^ The presence of lung metastases can only be revealed when the growth of the primary tumor is controlled. Thus, mice carrying primary subcutaneous tumors that are irradiated with 25 Gy will survive for a sufficient length of time (20–24 days after irradiation) before they exhibit clinical signs of lung metastases at which time they are sacrificed for assessment of lung disease. For this reason, a group of mice received an ablative dose of 25 Gy IR alone. This group was then used as a time-matched control, and lungs were excised and metastatic burden assessed when the majority of DSR-6434/15 Gy samples were removed (day 24). Mice that received 25 Gy had a metastases score of 4 ± 0.5; whereas those that received DSR-6434/15 Gy IR had a significantly lower metastases score of 1.2 ± 0.2 (Fig. 5*c*; *p* < 0.01).

## Discussion

Our data demonstrate that the novel compound DSR-6434 is a selective TLR7 agonist capable of activating T, B, NKT and NK cell populations and inducing the expression of IP-10 and IFNα following intravenous administration. Furthermore, our data demonstrate that while having no direct antitumor activity, DSR-6434 when administered in combination with IR was well tolerated, improved survival and induced long-term immunological memory in tumor bearing mice.

Topically administered TLR7 agonists such as imiquimod (Aldara, Graceway Pharmaceuticals) have proven effective for the treatment of dermatological malignancy, and more recently have demonstrated encouraging results in a phase II study involving topical imiquimod in combination with IR for the treatment of cutaneous metastases in patients with breast cancer.^30^ In these settings, the TLR7 agonist can be applied directly to the tumor cells, and may therefore directly affect the tumor microenvironment ultimately leading to activation of tumor antigen-specific CD8^+^ T cells. For the treatment of noncutaneous tumors, systemic administration of a TLR7 agonist may be required to stimulate an immune response with the capacity to modify the tumor-associated milieu and/or facilitate T-cell priming through activation of APCs. In contrast to the success of topically administered TLR7 agonists such as imiquimod, few trials have been conducted with systemically administered agents.

The precise mechanism by which TLR7 agonism enhances the antitumor immune response is unclear. TLR7 agonists have been shown to promote DC maturation, expression of tumor-associated antigen (TAA) and improved T-cell priming in a vaccination setting.^31^,^32^ However, agonism of TLR7 may also promote antitumor T-cell responses through the induction of multiple type-1 cytokines such as IFNα, IFNγ and IL-12^23^ (Fig 1*c* and Supporting Information Figs. 1 and 2). Despite this, TLR7 agonism using DSR-6434 as a monotherapy may not be sufficient to prime durable and efficacious CD8+ T-cell responses in certain animal models. This was reflected in our data since tumor resolution did not occur in any mice treated using this regime. Murine cell lines have different intrinsic levels of immunogenicity when implanted into syngeneic mouse strains. The CT26 model is considered to be more immunogenic^33^ than the KHT model,^29^ which may explain why there is a modest but significant effect of DSR-6434 monotherapy when treating mice implanted with CT26 but not KHT tumors. However, using models of lymphoma we have recently published that combination therapy with a systemic TLR7 agonist and local IR leads to the induction of a durable CD8^+^ T-cell response that mediates antitumor activity and increases survival.^14^

IR-induced cell death can be immunogenic through the upregulation of class I MHC on tumor cells and by cell surface expression and/or release of distinct DAMPs, leading to the activation of APCs and induction of tumor antigen-specific T-cell responses^5^,^6^,^34^. However, it is unclear how frequently this occurs in the clinic and indeed whether the induction of an antitumor immune response is partly responsible for the efficacy of this standard of care treatment. Data from vaccination studies clearly demonstrate the capacity of irradiated tumor cells to prime systemic immune responses that are protective against subsequent tumor challenge.^6^ In contrast, the treatment of established tumors in syngeneic models with IR alone is rarely capable of generating durable tumor antigen-specific immune responses.^10^,^11^ Several studies (reviewed in ref.^35^) have characterized the immuno-suppressive environment, which is thought to favor angiogenesis and potentially provide mitogenic stimuli within established tumors. It is likely that this microenvironment fosters immunological anergy to the potentially immunogenic cell death elicited following treatment with IR. Our preclinical data in mice bearing established CT26 or KHT tumors is in keeping with this hypothesis as despite an influx of CD8^+^ T cells following treatment with IR, no mice underwent complete response following treatment with IR alone.

Despite this, our data also support the concept that the induction of immunogenic cell death using IR in combination with an immunological adjuvant, such as DSR-6434, can elicit durable antitumor responses. Our results clearly demonstrate that once-weekly dosing of DSR-6434 greatly potentiates IR-induced tumor growth delay both in immunogenic CT26 and nonimmunogenic KHT tumor models, and resulted in complete tumor regression and LTS in 55% of CT26 tumor-bearing mice. Unlike imiquimod,^36^ the antitumor mechanism of action of DSR-6434 did not include a direct effect on tumor cell proliferation at pharmacologically relevant concentrations, nor did it increase sensitization to radiation *in vitro*, suggesting a mechanism of action involving host rather than tumor cells. The importance of CD8^+^ T cells in TLR7/IR combination therapy has been highlighted previously in lymphoma models, where the addition of the TLR7/8 agonist R-848 was ineffective at potentiating the effect of IR in CD8^+^ cell-depleted mice.^14^ Our data demonstrate that the combination of DSR-6434 with IR leads to the generation of memory CD8^+^ T cells using the CT26 model, which expresses the H2-Ld-restricted epitope, AH1; a peptide derived from an endogenous retroviral gene product; gp70.^37^ However, DSR-6434/IR therapy did not alter the infiltration of CD8^+^ T cells into the tumors, relative to IR monotherapy, suggesting a qualitative rather than quantitative enhancement of antitumor immunity.

In addition to enhancing growth delay of primary tumors following treatment with IR, combination with DSR-6434 also reduced the incidence of spontaneous lung metastases in the KHT sarcoma model. A phenomenon termed the abscopal effect has been previously described as the eradication of tumor cells from distant metastatic sites outside the field of radiation.^38^ Data suggest that the effect is mediated by a systemic adaptive immune response following IR that may be potentiated with the addition of immuno-stimulatory agents.^10^,^39^ Since KHT cell shedding from the primary tumor occurs within the first 7 days following implantation,^40^ and irradiation of tumors occurred around day 10, when tumors were 200 mm^3^, the reduction in lung metastases in the DSR-6434/IR combination group may be attributed to the abscopal effect. It is therefore also likely that the CD8^+^ T cells generated following IR/DSR-6434 treatment are specific for TAAs present not only on the irradiated tumor cells, but also those metastatic tumors developing in the lung that are not irradiated. Interestingly, this suggests that the immune response is to endogenous TAAs expressed by the KHT cells and not due to the expression of novel peptides as a result of IR-induced DNA mutation and aberrant protein production.

Around 50% of cancer patients receive radiotherapy as part of their treatment regime; however, many patients suffer from disease recurrence of metastasis.^4^ Novel approaches that aim to combine cytoreductive therapies with immuno-potentiating agents such as immuno-stimulants or inhibitors of immunological checkpoints may improve patient outcome. This study supports the rationale for combining systemically administered TLR7 agonists in combination with IR to improve the local and metastatic control of solid tumors, and warrants clinical evaluation.

## Abbreviations

APC: antigen presenting cell
DAMP: damage-associated molecular pattern
DC: dendritic cell
HMGB1: high mobility group box 1
IFN: interferon
IL: Interleukin
IR: ionizing radiation
IRF-7: interferon regulatory factor-7
LTS: long-term surviving
TAA: tumor-associated antigen
TGF-β: transforming growth factor-β
TLR-7: Toll-like receptor 7
