# Supplementary material for: A novel systemically administered toll-like receptor 7 agonist potentiates the effect of ionizing radiation in murine solid tumor models
Source: Int J Cancer. 2014 Jan 17;135(4):820–9. doi: 10.1002/ijc.28711 (PMC4286010; doi:10.1002/ijc.28711)

Supplementary Methods

In vitro analysis of cytokine induction from splenocytes

For the measurement of cytokine induction *in vitro*, splenocytes taken from wild type (+/+; n=3) or TLR7 knockout (-/-; n=3) mice were treated with 0-2000 nM of DSR-6434 or 2000 nM DSR-6434, respectively. Twenty four hours after treatment, IP-10, IL-12(p70), IFN-γ, KC and TNF-α expression was measured by Milliplex immunoassay in accordance with the manufacturer's protocol (Millipore). Fluorescence was measured using a Luminex 2000 (Luminex Corporation), and mean concentrations + SEM were plotted.

Analysis of cytokine levels in plasma

BALB/c wild-type (wt) and TLR7 -/- mice were dosed intravenously with 0.1 mg/kg DSR-6434 in Saline (adjusted to pH5). Blood samples were taken at 2, 4, 8, 12 and 24 h after administration, and plasma samples were collected following centrifugation at 2486 x g for 10 min at 4 °C. IFNγ, TNFα and KC were analyzed by Milliplex assay (Millipore) according to manufacturer's instructions.

Immunohistochemistry (IHC)

Air-dried cryosections of 8 μm thickness were fixed in 4 % (w/v) paraformaldehyde in PBS. Endogenous peroxidase activity and non-specific antibody binding were blocked by incubating with peroxidase block for 15 min and Rodent Block for 20 min (both MenaPath, UK). A CD8α primary antibody (0.83 μg/mL; BD Pharmingen) was applied to sections for 60 min. Rat IgG2a control (eBioscience) was included, and spleen sections were used as positive controls. Rat Probe secondary reagent and Rat HRP Polymer (both MenaPath) were used according to manufacturer's instructions. DAB chromogen (DAKO) was applied for 10

min, followed by counterstaining with Gills hematoxylin for 1 min and dehydration before permanent mounting. Slides were scanned and whole sections were analyzed using Definiens Tissue Studio. Percentage of positive pixels was plotted as mean + SEM for 5 tumors per treatment group.

Supplementary figure legends

S.1: Cytokine induction 24 h after administration of DSR-6434. Splenocytes isolated from BALB/c mice either wildtype (+/+; n=3) or knockout (-/-; n=3) for TLR7 were treated with a range of DSR-6434 concentrations (0-2000 nM), or 2000 nM, respectively. 24 h after treatment, IP-10, IL-12(p70), IFN-γ, KC and TNF-α levels were analyzed using Milliplex immunoassay. Plotted are mean concentrations + SEM.

S.2: Plasma levels of cytokines following DSR-6434 administration. BALB/c TLR7 wt and TLR7 -/- mice received DSR-6434 (i.v.) and plasma samples were analysed for the presence of IFNγ, TNFα and KC at time-points between 2 and 24 h post-treatment. Plotted are means ± SEM of 3 mice per group. * $P<0.05$ when comparing WT with -/- mice (two-tailed Student's *t* test).

S.3: Representative histograms for CD69 expression presented in Fig. 1D. Splenocytes were harvested from CT26 and KHT tumor-bearing BALB/c (left) and C3H (right) mice, respectively, 4 h after i.v. injection with saline (black histogram) or 0.1 mg/kg DSR-6434 (grey histogram). CD69 expression as MFI was analyzed by flow cytometry.

S.4: Mouse weight following DSR-6434 administration. Tumor-bearing BALB/c (A) and C3H mice (B) received i.v. injections of 0.1 mg/kg DSR-6434 on days indicated by dotted lines and weight was monitored daily. Weight was also monitored following combination treatment with 0.1mg/kg DSR-6434 once weekly, with 5 daily doses of 2Gy IR (CT26; C) or 15Gy IR (KHT; D) beginning 4 h after the first dose of DSR-6434.

S.5: Efficacy of DSR-6434 administered at 0.01 mg/kg in CT26 tumor-bearing mice. Mice received saline (n=5), 0.01 mg/kg DSR-6434 (n=5), 5 fractions of 2 Gy IR (n=4) or a combination (n=4). A: Tumor growth curves showing mean ± SEM until the first tumor from each group is removed. B: Survival curves for cohorts above. Surviving mice are those that have not reached RTV4.

S.6: Growth delay data for CT26 (A) and KHT (B) tumors. Shown are mean (±SEM) RTV4 values pooled from two identical experiments depicted in Fig. 3 and Fig. 5. Treatment groups were saline or 0.1 mg/kg DSR-6434 (i.v.) once weekly as monotherapy or in combination with 5 daily doses of 2Gy IR (CT26) or 15 Gy single dose IR (KHT) beginning 4 h after the first i.v. dose. Long-term surviving mice are excluded from data in this table. ** $P<0.01$ relative to saline control; *** $P<0.001$ relative to saline control; +++ $P<0.001$ relative to either monotherapy.

S.7: Local tumor therapy with IR leads to enhanced $CD8^+$ T cell infiltration that is not affected by combination therapy with DSR-6434. $CD8^+$ cell infiltration was measured in tumors treated with 5 fractions of 2 Gy IR with or without 0.1 mg/kg DSR-6434 in comparison to untreated (NT) controls grown to 400 mm^3. Whole section analysis of infiltrate was generated using Definiens Tissue Studio. A: Data plotted as the percentage of pixels positive for CD8. Means + SEM are plotted for 5 mice per group. * $P<0.05$ relative to untreated control; n/s = not significant. B: Representative microphotographs for each treatment group. Brown DAB staining indicates $CD8\alpha^+$ cells.

Supplementary figures

Supplementary Fig. 1

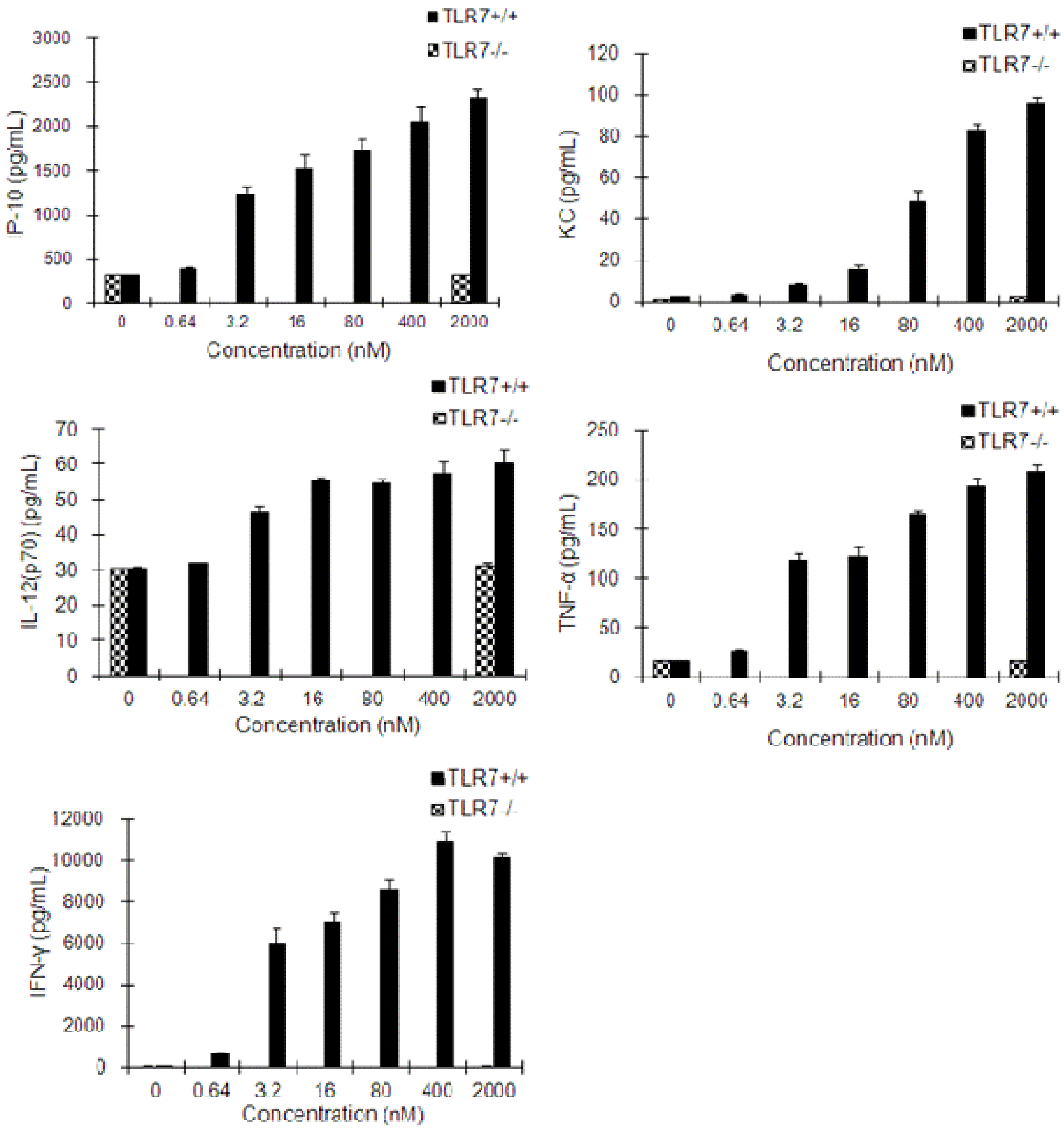

Supplementary Fig. 2

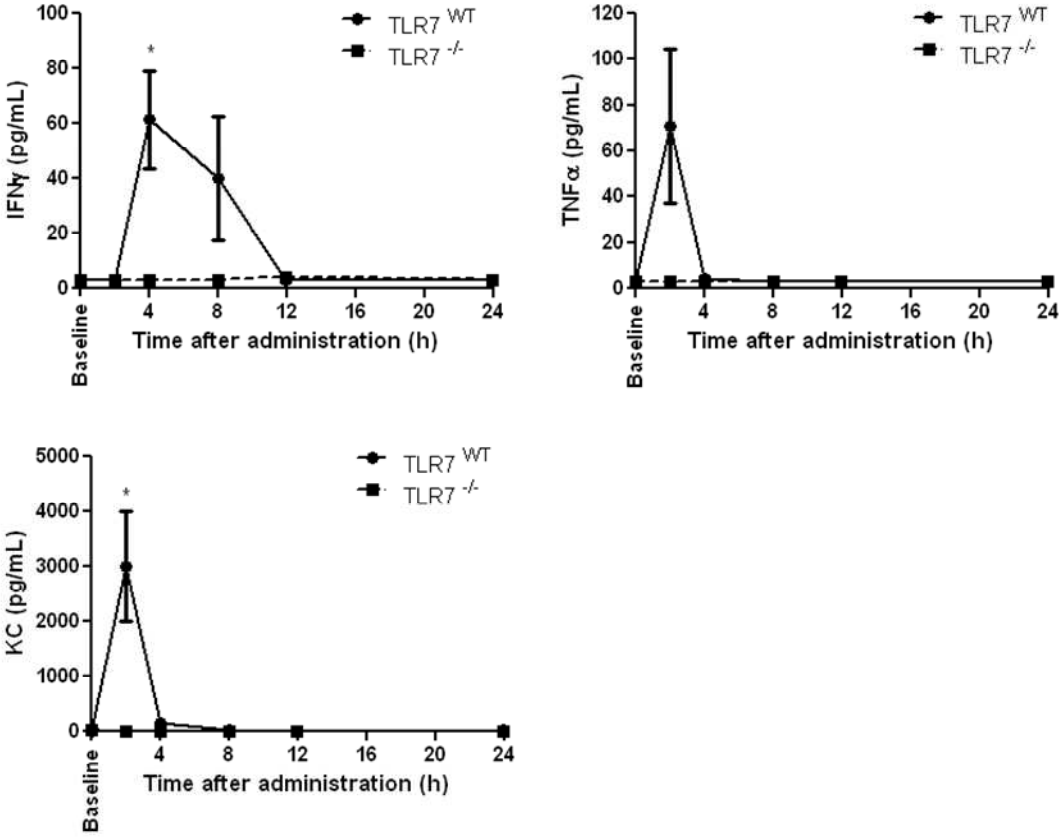

Supplementary Fig. 3

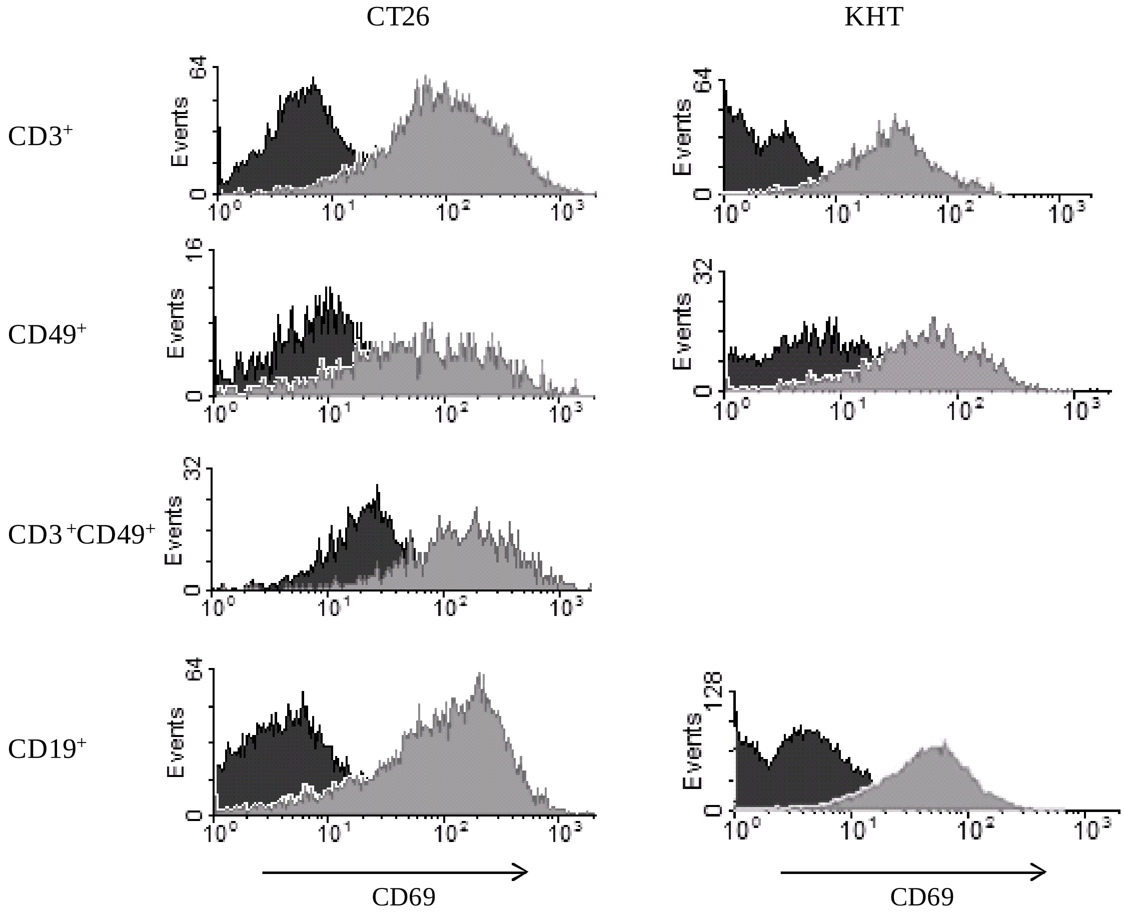

Supplementary Fig. 4

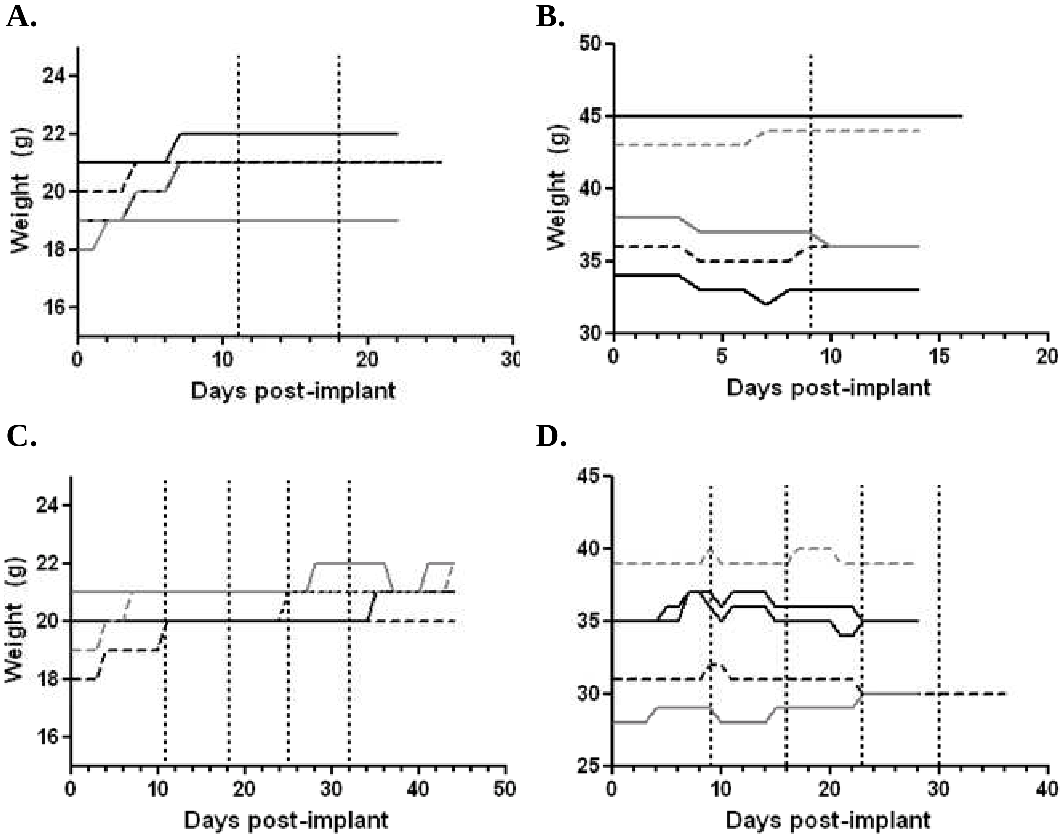

Supplementary Fig. 5

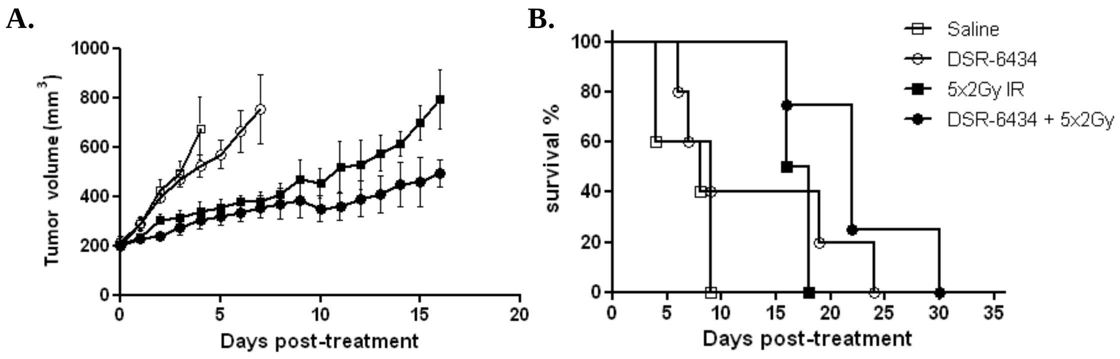

Supplementary Fig. 6

CT26		KHT	
**Treatment**	**Days to reach RTV4 (mean ± SEM)**	**Treatment**	**Days to reach RTV4 (mean ± SEM)**
**Saline**	6 ± 0.42	**Saline**	4.67 ± 0.33
**DSR-6434**	9.55 ± 1.3 **	**DSR-6434**	4.67 ± 0.87
**5x2Gy IR**	23.94 ± 3.75 ***	**15Gy IR**	16.4 ± 0.67 **
**DSR-6434 + 5x2Gy IR**	40.7 ± 3.36 *** +++	**DSR-6434 + 15Gy IR**	23.89 ± 1.54 *** +++

Supplementary Fig. 7

A.

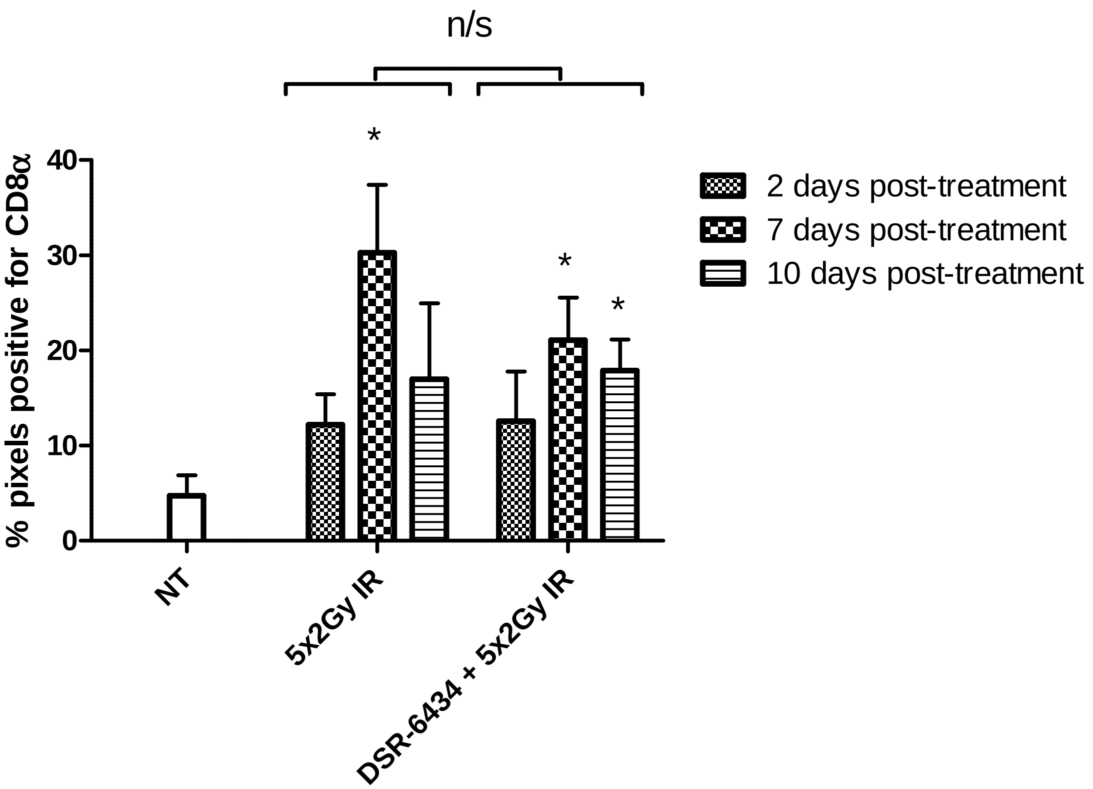

B.

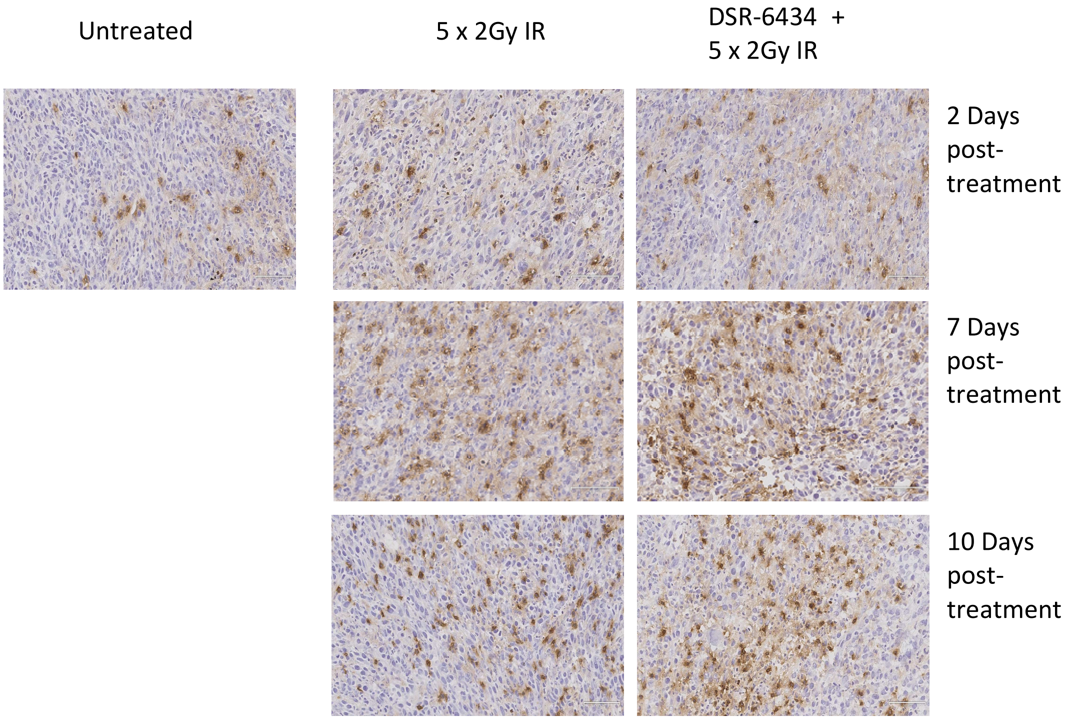

Supplement: Supplementary file 1 [file ijc0135-0820-sd1.pdf]
